# Coagulation profile in patients undergoing video-assisted thoracoscopic lobectomy: A randomized, controlled trial

**DOI:** 10.1371/journal.pone.0171809

**Published:** 2017-02-15

**Authors:** Thomas Decker Christensen, Henrik Vad, Søren Pedersen, Kåre Hornbech, Nora Elisabeth Zois, Peter B. Licht, Mads Nybo, Anne-Mette Hvas

**Affiliations:** 1 Department of Cardiothoracic and Vascular Surgery & Institute of Clinical Medicine, Aarhus University Hospital, Aarhus, Denmark; 2 Department of Anesthesiology and Intensive Care & Institute of Clinical Medicine, Aarhus University Hospital, Aarhus, Denmark; 3 Department of Cardio-thoracic Surgery, Rigshospitalet, Copenhagen University Hospital, Copenhagen, Denmark; 4 Department of Clinical Biochemistry, Rigshospitalet, Copenhagen University Hospital, Copenhagen, Denmark; 5 Department of Cardiothoracic and Vascular Surgery, Odense University Hospital, Odense, Denmark; 6 Department of Clinical Biochemistry, Odense University Hospital, Odense, Denmark; 7 Department of Clinical Biochemistry & Institute of Clinical Medicine, Aarhus University Hospital, Aarhus, Denmark; Maastricht University Medical Center, NETHERLANDS

## Abstract

**Background:**

Knowledge about the impact of Low-Molecular-Weight Heparin (LMWH) on the coagulation system in patients undergoing minimal invasive lung cancer surgery is sparse. The aim of this study was to assess the effect of LMWH on the coagulation system in patients undergoing Video-Assisted Thoracoscopic Surgery (VATS) lobectomy for primary lung cancer.

**Methods:**

Sixty-three patients diagnosed with primary lung cancer undergoing VATS lobectomy were randomized to either subcutaneous injection with dalteparin (Fragmin^®^) 5000 IE once daily or no intervention. Coagulation was assessed pre-, peri-, and the first two days postoperatively by standard coagulation blood test, thromboelastometry (ROTEM^®^) and thrombin generation.

**Results:**

Patients undergoing potential curative surgery for lung cancer were not hypercoagulable preoperatively. There was no statistically significant difference in the majority of the assessed coagulation parameters after LMWH, except that the no intervention group had a higher peak thrombin and a shorter INTEM clotting time on the first postoperative day and a lower fibrinogen level on the second postoperative day. A lower level of fibrin d-dimer in the LMWH group was found on the 1. and 2.postoperative day, although not statistical significant. No differences were found between the two groups in the amount of bleeding or number of thromboembolic events.

**Conclusions:**

Use of LMWH administered once daily as thromboprophylaxis did not alter the coagulation profile *per se*. As the present study primarily evaluated biochemical endpoints, further studies using clinical endpoints are needed in regards of an optimized thromboprophylaxis approach.

## Introduction

Cancer is generally associated with hypercoagulability [1] and thereby an increased risk of venous thromboembolic events (VTE). The risk of VTE may be due to increased activation of the coagulation system, which brings the patient into a hypercoagulable state [2].

The overall evidence for using low-molecular-weight heparin (LMWH) in surgical cancer patients is relatively weak; only one randomized, controlled trial has been published since 1996, where patients undergoing colorectal surgery either received LMWH or placebo/no intervention [3]. The authors found no difference between the two regimes. Yet, there are many observational studies published favouring thromboprophylaxis in cancer patients undergoing surgery, and LMWH is generally recommended [3]. However, the use of LMWH results in increased cost, an increased risk of bleeding, logistical difficulties, and pain due to the injection.

LMWH thromboprophylaxis is also widely recommended for patients undergoing lung cancer surgery [4, 3]. However, only a very limited number of studies have investigated LMWH’s impact on the coagulation system in lung cancer surgery, and no studies have investigated it in minimal invasive surgery.

Attaran et al [5] used thromboelastography (TEG^®^) and found that lung cancer patients undergoing surgery were not hypercoagulable compared with patients undergoing operation for benign diseases. They showed that LMWH administration once or twice daily did not provide sufficient thromboprophylaxis and advocated for screening of patients using TEG^®^ and ensuring adequate thromboprophylaxis in hypercoagulable patients only.

Thromboelastometry (ROTEM^®^), thrombin generation and standard coagulation parameters assess altogether the total haemostatic capacity quantifying both global and dynamic coagulation parameters [6, 7].

It seems important to assemble further knowledge in order to target and optimize the thromboprophylaxis given, e.g. in terms of type of medication, screening for hypercoagulation, influence of using a minimal invasive surgical approach, timing and length of prophylaxis.

We hypothesized that LMWH administered once daily would reduce the ROTEM, EXTEM clotting time following surgery in patients undergoing Video-Assisted Thoracoscopic Surgery (VATS) lobectomy for primary lung cancer.

Thus, the aim of this study was therefore to assess the effect of LMWH on the coagulation system in patients undergoing Video-Assisted Thoracoscopic Surgery (VATS) lobectomy for primary lung cancer.

## Materials and methods

Patients referred for lobectomy to Aarhus University Hospital, Rigshospitalet (University Hospital of Copenhagen) or Odense University Hospital, Denmark, in the period from March 2013 to April 2015 was screened for eligibility.

Inclusion criteria were: (1) Diagnosed with primary lung cancer with a preoperative stage IA-IB; (2) Surgery with expected lobectomy or bi-lobectomy using VATS; (3) Willingness to participate and ability to give informed oral and written consent; and (4) > 18 years of age at time of randomization. Exclusion criteria were: (1) Thromboembolic event (either arterial or venous) within the past three months; (2) Pregnant or lactating; (3) Treatment with vitamin K-antagonist or a non-vitamin K antagonist oral anticoagulant; or (4) Treatment with a platelet inhibitor if this was not paused for a minimum of 5 days (aspirin, clopidogrel or ticagrelor) or 7 days (prasugrel). No patients had received neoadjuvant chemo- and/or radiation therapy prior to surgery.

The patients were included after oral and written consent. The protocol for the study complied with the Helsinki II declaration and was approved by the local scientific ethical committee (File number: 1-10-72-364-12) and The Danish Data Protection Agency. The study was conducted according to Good Clinical Practice standards and was monitored and approved by the Good Clinical Practice unit, Aarhus University Hospital, Aarhus, Denmark. The trial was registered at ClinicalTrials.gov (Identifier: NCT01741506) and at EudraCT no. 2012-002409-23.

### Patients

Sixty-three patients undergoing VATS lobectomy were included. Patients were randomly assigned to LMWH or no intervention using a computerized prospective randomization schedule. Randomization was performed in blocks with various sizes in numbers of 2, 4 and 6 without blinding of allocation.

### Intervention

Patient randomized to LMWH received dalteparin (Fragmin^®^) (Pfizer Inc., New York, USA). It was provided as subcutaneous injections at a dose of 5000 IE once daily, starting the day before surgery (approximately 12 hours before surgery), and then administered daily at 22:00 (10:00 PM) until the patients were discharged. The patients randomized to no intervention received no LMWH.

All operations were performed in general anaesthesia with propofol and fentanyl. The VATS approach used has previously been described in details [8]. Briefly, an anterior approach with one incision and two port assist incisions were performed, and one chest tube was placed. All patients were extubated immediately after surgery and mobilized starting on the day of surgery.

### Observation period and blood analyses

Blood samples were obtained and analysed at the following four time-points: 1) Preoperatively; the day before surgery (and before LMWH potentially were given); 2) Perioperatively at the time of stapling the bronchus; 3) Postoperatively 08:00 AM at day 1; and 4) Postoperatively 08:00 AM at day 2. LMWH was therefore given approximately 10 hours before blood samples were taken

The first 2 ml of blood was discarded before drawing blood into tubes containing sodium citrate for ROTEM^®^ analyses, thrombin generation and standard coagulation analyses including: Activated partial thromboplastin time (APTT), International Normalized Ratio (INR), fibrinogen (functional, Claus method), fibrin d-dimer, thrombin time, platelet count and factor (F) VIII:Clot. Blood for ROTEM^®^ analyses were left at room temperature for 30 minutes before processing, whereas remaining analyses were done either as routine analyses or blood samples were centrifuged at 2800 g for 25 minutes and plasma was stored in aliquots at—80°C.

Regarding thromboelastometry (ROTEM^®^, Tem International GmbH, Munich, Germany), three standard assays were performed: INTEM, EXTEM, and FIBTEM. We obtained the dynamic parameters of clot initiation (clotting time: CT, seconds (s)) and clot propagation (maximum velocity of clot formation: MaxVel, mm x 100/s, time to maximum velocity: tMaxVel, s), and whole blood clot strength was assessed by maximum clot firmness (MCF, mm x 100).

Thrombin generation was evaluated by calibrated automated thrombogram (CAT; Thrombinoscope BV, Maastricht, the Netherlands) using platelet-poor plasma. The following parameters were analysed: Lag-time until initial thrombin generation (minutes), maximum concentration of thrombin (peak, nM), time to peak (ttpeak, minutes), and the endogenous thrombin potential (ETP, nM x minutes).

Reference values for the ROTEM^®^ was calculated based on data obtained from 73 healthy individuals previously published [9], while reference values for thrombin generation was obtained from 32 individuals published by Collins PW et al. [10].

APTT (Platelin LS, Organon, Munich, Germany), INR (Owren’s PT-reagent, MediRox), fibrinogen (Clauss method, Siemens Dade reagent), thrombin time (Siemens Test Thrombin), fibrin d-dimer (Siemens INNOVANCE^®^ D-Dimer reagent), and Anti-Xa (Berichrom heparin, Siemens) were analysed employing the CS2100i (Sysmex, Kobe Japan). Factor VIII:Clot was analysed by ACL-TOP (Istrumental Laboratory, Bedford, MA, USA)

Preoperative data in terms of clinical characteristics was collected systematically from medical records. Furthermore, peri- and postoperative data (e.g. operating time, bleeding during surgery, total drain loss, VTE and adverse events) was collected prospectively. VTE were captured through clinical assessment and by reviewing medical records. If VTE was suspected, an ultrasound was performed.

All patients were contacted by phone 30 days after the operation and had also their medical records reviewed.

### Statistical analyses, endpoints and sample size

Baseline data, peri- and postoperative characteristics were presented using descriptive statistics. Results of the coagulation analyses were tested for normal distribution and hence presented as either mean and standard deviation (SD) or median and 95% confidence interval (CI) or as minimum to maximum values. Normally distributed data was compared using Student’s unpaired t-tests, while non-normally distributed data was compared using Mann-Whitney U-tests.

Microsoft^®^ Excel^®^ for Mac 2011 (Microsoft^®^, Seattle, USA) and GraphPad Prism for Mac (GraphPad Software, Inc., CA, USA) was used for the statistical analyses.

The study was primarily an explorative study, and the sample size is therefore associated with some uncertainty. We based it on the ROTEM^®^ EXTEM: CT (clotting time). We were not able to find any studies investigating ROTEM^®^ in thoracic surgical patients undergoing operations for lung cancer. In a healthy population the mean is 60 sec with a SD of 25 sec. [9]. The minimal relevant difference in terms of clotting was designated to 20 sec. In order to detect this difference with a type I error of 0.05 and 90% power, 27 patients were needed in each group. Due to the anticipated missing values, 30 patients in each group were considered appropriate.

Analysis was done using the intention to treat principal.

## Results

**Fig 1:**
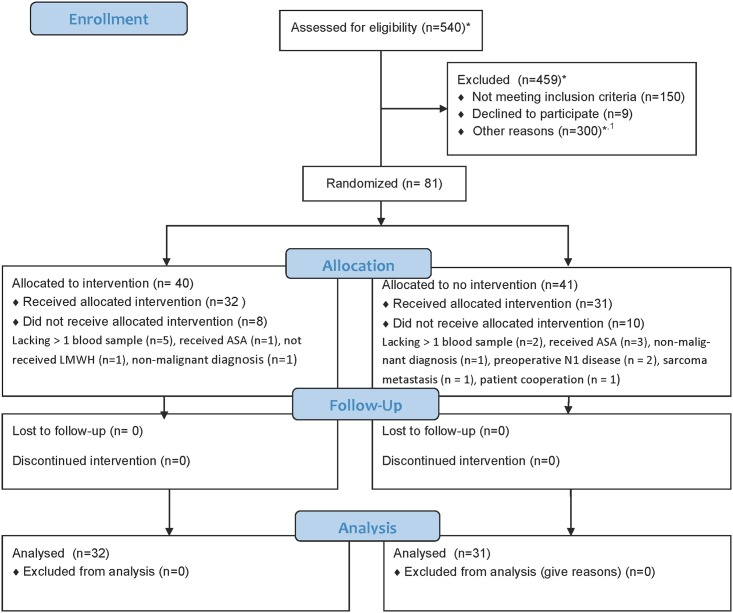
Trial flowchart for patients planned for Video-Assisted Thoracoscopic Surgery (VATS) lobectomy for primary lung cancer. *: Estimated with some uncertainty. ^**1**^**: This includes: 1) Patients operated on Thursday and Fridays; 2) No specialized laboratory technicians available; 3) Not practical possible to get permission from the patient to participate; 4) Other logistic problems.** Abbreviations: n/N: numbers; ASA: Acetylsalicylic acid (aspirin); LMWH: Low-Molecular-Weight Heparin; NSAID: Non-Steroid Anti Inflammatory Drug.

Fig 1 displays the trial flowchart. A total of 81 VATS-patients were randomized; 40 patients to the LMWH arm and 41 patients to the no intervention group. In the LMWH arm 8 patients were excluded due to: lacking > one blood sample (n = 5), received acetylsalicylic acid (ASA) in terms of aspirin (n = 1), did not receive LMWH (n = 1), non-malignant diagnosis (n = 1). A total of 32 patients were therefore included in the LMWH arm, of which 2 patients had been given Non-Steroid Anti Inflammatory Drug (NSAID). Additionally, 1 patient was converted to an open procedure.

In the no intervention arm, 10 patients were excluded due to: lacking > one blood sample (n = 2), received ASA (n = 3), received LMWH (n = 1), preoperative N1 disease (n = 1), sarcoma metastasis (n = 1), non-malignant diagnosis (n = 1) and lack of patient cooperation (n = 1). A total of 31 patients were therefore included in the no intervention arm, of which 3 had been given NSAID. Additionally, 3 patients were converted to an open procedure. Thus, a total of 63 patients were included in the analyses.

Table 1 shows baseline/preoperative characteristics. As expected there was no statistically significant difference due to the randomization (p-values not shown). The peri- and postoperative data are displayed in Table 2. There was no difference in terms of perioperative bleeding or in the total amount of fluid drained. As expected we found a limited number of major adverse events (death, major bleeding and thromboembolic events). However, there was a tendency towards more adverse events in the no intervention group (3 in the LMWH group and 5 in the no intervention group). There was one death in LMWH group and two incidents of apoplexia cerebri in the no intervention group.

**Table 1:** Baseline data (preoperative) from 63 patients planned for Video-Assisted Thoracoscopic Surgery (VATS) lobectomy for primary lung cancer.

Characteristic	LMWH group (N = 32)	No intervention group (N = 31)
**Age (years)**	69.8 (7.3)	67.0 (10.3)
**Sex (n female/n male)**	15/17	20/11
**Non smoker/ex-smoker/ active smoker, n**	1/24/7	1/19/11
**Pack years of smoking**	30.9 (23.3)	29.8 (19.9)
**FEV 1 (% of expected)**	83.4 (27.0)	91.1 (20.0)
**DLCO (% of expected)**	69.0 (24.2)	68.8 (16.7)
**BMI**	25.8 (4.0)	24.7 (3.6)
**Co-morbidity**^1^**, n:**		
Diabetes	3	2
Hypertension	13	12
Hyperlipidemia	9	10
Cardial and/or vascular disease	8	4
Previous malignant disease	6	8
**ASA prescribed, n (%)**	7 (22)	6 (19)
**Laboratory analyses reference interval):**		
B—Haemoglobin (women: 7.3–9.5 mmol/L; men: 8.3–10.5 mmol/L)	8.6 (0.9)	8.5 (0.7)
B—Leukocytes (3.5–10.0 x10^9^/L)	7.9 (1.9)	7.5 (2.0)
P—Creatinine (45–105 μmol/L)	73 (20)	72 (18)
B—Platelet count (145–400 x10^9^/L)	289 (69)	287 (85)
P—CRP (< 8 mg/L)	7.7 (12.3)	4.0 (4.3)
INR (< 1.2)	1.0 (0.1)	1.0 (0.1)

All values are provided either as mean (standard deviation) or numbers (percentage).

^1^Defined as the patient being in medical treatment for the disease.

Abbreviations: B: Blood; N/n: Numbers; P: Plasma; ASA: Acetylsalicylic acid (aspirin); BMI: Body Mass Index; CRP: C-reactive protein; DLCO: Diffusion Capacity of the Lung for Carbon Monoxide; FEV: Forced Expiratory Volume; INR: International Normalized Ratio; LMWH: Low-Molecular-Weight Heparin.

**Table 2:** Peri- and postoperative data from 63 patients planned for Video-Assisted Thoracoscopic Surgery (VATS) lobectomy for primary lung cancer.

Characteristic	LMWH group (N = 32)	No intervention group (N = 31)	p-value
**Type of lobectomy**			
Right Upper Lobe	10	10	-
Right Middle Lobe	1	1	-
Right Lower Lobe	8	10	-
Left Upper Lobe	9	7	-
Left Lower Lobe	4	3	-
**Operating time (h:mm)**	2:29 (0:47)	2.31 (0:47)	0.43
**Bleeding/drainage during surgery (mL)**	100 (0–300)	100 (0–2000)	0.96
**Use of inotropes (n of patients)**^1^	17	15	-
**Re-operated (n)**	1	1	-
**Total amount of fluid in the chest drain (mL)**	760 (50–4356)	850 (50–6135)	0.91
**Complications**^2^ **(n)**	2^3^	5^4^	-
**VTE events**	0	0	-
**Death (n)**	1^5^	0	-
**Total length of stay (days)**	6.3 (3.0–32.4)	5.5 (3.2–20.0)	0.31
**Type of cancer**			
Adenocarcinoma	22	23	-
Squamous cell carcinoma	8	4	-
Carcinoid (all types)	0	1	-
Others^6^	2	3	-
**Pathological staging**			-
Stage IA+B	28	27	-
Stage IIA+B	4	4	-
**Microscopically free resection margins (R0)**	32	31	-

Values for the operating time are provided as mean (standard deviation) and tested using a t-test. The other values are displayed as median (minimum to maximum) since data was not normally distributed. Difference was tested using Mann-Whitney U-test.

^1^Predominantly small doses of methaoxidrin or efedrin.

^2^Includes myocardial infarction, apoplexia cerebri and atrial fibrillation.

^3^Both were atrial fibrillation.

^4^Apoplexia cerebri (n = 2) and atrial fibrillation (n = 3).

^5^Died postoperatively (probably due to bleeding (tamponade)).

^6^Includes small cell carcinoma, neuroendocrine and sarcotomoid tumor.

Abbreviations: N/n: Numbers; LMWH: Low-Molecular-Weight Heparin; H: Hours; mL: Milliliter; Mm: Minutes; VTE: Venous Tromboembolic Events.

Patients experiencing atrial fibrillation was converted to sinus rhythm using amiodarone, and no additionally antithrombotic treatment were provided.

The results of the standard coagulation blood tests are shown in Table 3, ROTEM^®^ results in Table 4, and thrombin generation in Table 5. Overall, patients did not show a hypercoagulable state prior to surgery. However, 13 patients in the LMWH group had FIBTEM maximum clot firmness (MCF) above 20 mm compared with 15 patients in the no intervention group.

**Table 3:** Conventional coagulation tests among 63 patients undergoing Video-Assisted Thoracoscopic Surgery (VATS) lobectomy for primary lung cancer.

Conventional coagulation tests (reference interval)	Preoperative			Perioperative			First postoperative day			Second postoperative day		
	+ LMWH	No LMWH	p-value	+ LMWH	No LMWH	p-value	+ LMWH	No LMWH	p-value	+ LMWH	No LMWH	p-value
APTT (28–38 sec)	31 (4)	30 (4)	0.79	30 (30–34)	32 (31–36)	0.33	32 (31–36)	30 (29–33)	0.05	33 (5)	32 (5)	0.37
INR (< 1.2)	1.0 (1.01–1.06)	1.0 (0.98–1.02)	0.05	1.1 (1.06–1.14)	1.06 (1.03–1.08)	0.20	1.10 (1.10–1.18)	1.10 (1.09–1.16)	0.63	1.09 (1.06–1.13)	1.10 (1.06–1.15)	0.81
Fibrinogen (5–12 μmol/L)	10 (9–11)	10 (9–11)	0.76	8 (8–9)	8 (8–9)	0.58	11 (10–12)	10 (10–12)	0.25	15 (2)	13 (3)	0.02
Platelet count (145–400 x 10^9^/L)	280 (61)	281 (89)	0.99	225 (52)	228 (59)	0.84	231 (48)	234 (61)	0.82	243 (70)	225 (64)	0.38
Thrombin time (< 21 sec)	17 (17–18)	17 (16–17)	0.75	17 (17–19)	18 (17–18)	0.96	16 (16.0–17)	16 (16–17)	0.37	15 (2)	16 (1)	0.57
Fibrin d-dimer (< 0.50 mg/L FEU)	0.49 (0.45–1.03)	0.41 (0.47–1.03)	0.52	0.73 (0.50–1.56)	0.51 (0.47–1.04)	0.30	0.84 (0.57–2.64)	1.12 (1.19–2.98)	0.06	0.73 (0.72–1.15)	1.10 (0.88–2.27)	0.18
Factor VIII:Clot (0.66–1.55 kiu/L)	1.36 (1.18–1.50)	1.28 (1.23–1.51)	0.76	1.28 (1.16–1.47)	1.15 (1.07–1.26)	0.11	1.53 (1.55–1.81)	1.47 (1.41–1.67)	0.08	1.85 (1.77–2.08)	1.76 (1.63–1.96)	0.27

Data were tested for normal distribution using the D-Agostino & Pearson Omnibus normality test (alpha set to 0.05); if normally distributed, t-tests were used, while if non-normal distribution in one or both groups was found a non-parametric test (Mann-Whitney U test) was applied.

Normally distributed data are shown as means (standard deviations), whereas non-normally distributed data are shown as medians and (95% confidence intervals).

Abbreviations: APTT: Activated partial thromboplastin time; LMWH: Low-Molecular-Weight Heparin; INR: International Normalized Ratio.

**Table 4:** ROTEM^®^ results among 63 patients undergoing Video-Assisted Thoracoscopic Surgery (VATS) lobectomy for primary lung cancer.

ROTEM^®^ variables (reference interval)	Preoperative			Perioperative			First postoperative day			Second postoperative day		
	+ LMWH	No LMWH	p-value	+ LMWH	No LMWH	p-value	+ LMWH	No LMWH	p-value	+ LMWH	No LMWH	p-value
**EXTEM**												
CT (38–74 sec)	58 (54–61)	59 (54–65)	0.80	56 (52–58)	55 (53–64)	0.51	58 (55–60)	59 (52–63)	0.63	59 (55–62)	58 (53–67)	0.90
MaxVel (8–22 mm/min)	20 (4)	21 (5)	0.20	18 (4)	19 (6)	0.45	19 (18–20)	18 (18–21)	0.71	22 (5)	21 (5)	0.71
t,MaxVel (48–145 sec)	91 (26)	92 (26)	0.87	91 (25)	99 (28)	0.28	88 (21)	89 (30)	0.96	76 (74–99)	81 (78–102)	0.59
**INTEM**												
CT (129–181 sec)	165 (161–175)	165 (161–176)	0.66	170 (150–173)	169 (162–177)	0.67	167 (19)	156 (14)	0.01	166 (15)	1560 (15)	0.10
MaxVel (11–25 mm/min)	22 (4)	23 (5)	0.41	21 (4)	22 (5)	0.48	21 (19–25)	22 (20–23)	0.71	22 (4)	22 (5)	0.98
t,MaxVel (147–223 sec)	190 (185–203)	192 (188–204)	0.52	193 (179–197)	193 (186–203)	0.56	191 (21)	181 (18)	0.05	190 (18)	185 (21)	0.35
**FIBTEM**												
MCF (8–20 mm)	19 (18–24)	21 (19–25)	0.52	17 (15–21)	17 (16–21)	0.83	22 (20–24)	21 (19–24)	0.62	28 (6)	25 (6)	0.08

Data were tested for normal distribution using the D-Agostino & Pearson Omnibus normality test (alpha set to 0.05); if normally distributed, t-tests were used, while if non-normal distribution in one or both groups was found a non-parametric test (Mann-Whitney U test) was applied.

Normally distributed data are shown as means (standard deviations), whereas non-normally distributed data are shown as medians (95% confidence intervals).

Abbreviations: CT: Clotting Time; LMWH: Low-Molecular-Weight Heparin; MaxVel: Maximum Velocity; tMaxVel: Time to Maximum Velocity; MCF: Maximum Clot Firmness.

**Table 5:** Thrombin generation among 63 patients undergoing Video-Assisted Thoracoscopic Surgery (VATS) lobectomy for primary lung cancer.

Thrombin generation (reference interval^1^)	Preoperative			Perioperative			First postoperative day			Second postoperative day		
	+ LMWH	No LMWH	p-value	+ LMWH	No LMWH	p-value	+ LMWH	No LMWH	p-value	+ LMWH	No LMWH	p-value
Lag time (2.4 (0.9) min)	3.3 (3.0–3.6)	3.5 (3.1–3.9)	0.57	2.8 (2.7–3.2)	3.0 (2.8–3.5)	0.39	3.7 (3.5–5.1)	3.1 (3.1–3.9)	0.15	3.0 (3.2–5.1)	3.2 (3.1–3.9)	0.35
Peak thrombin (454 (100) nM)	220 (189–235)	215 (202–255)	0.53	241 (54)	239 (62)	0.86	173 (85)	221 (65)	0.02	258 (216–274)	259 (222–271)	0.75
ETP (1681 (281) nM x min)	1271 (1167–1410)	1294 (1264–1280)	0.26	1381 (214.1)	1381 (236.2)	0.98	1089 (899.4–1172)	1227 (753.7–2549)	0.07	1351 (1140–1390)	1236 (1152–1300)	0.07
Time to peak (4.2 (1.2) min)	6.7 (6.2–7.3)	6.8 (6.4–7.6)	0.78	5.9 (1.2)	6.2 (1.5)	0.39	6.9 (6.4–10.8)	6.4 (5.9–7.1)	0.12	5.5 (5.6–8.6)	5.6 (5.5–6.7)	0.48

Data were tested for normal distribution using the D-Agostino & Pearson Omnibus normality test (alpha set to 0.05); if normally distributed, t-tests were used, while if non-normal distribution in one or both groups was found a non-parametric test (Mann-Whitney U test) was applied.

Normally distributed data are shown as means (standard deviations), whereas non-normally distributed data are shown as medians (95% confidence intervals).

^1^Displayed as mean (standard deviation).

Abbreviations: LMWH: Low-Molecular-Weight Heparin; ETP: Endogenous Thrombin Potential

A lower level of fibrin d-dimer in the LMWH group was found on the 1. and 2.postoperative day, although not statistical significant.

The only statistically significant differences were that the no intervention group had a shorter INTEM CT on the first postoperative day compared to the LMWH group and a lower fibrinogen level on the second postoperative day and a higher peak thrombin as well.

The anti-Xa activity was analysed in the blood-samples taken perioperative and on the 1.postoperative day and 2.postoperative day for the patients randomized to LMWH. The results are presented in Table 6.

**Table 6:** Anti-factor Xa data from 32 patients planned for Video-Assisted Thoracoscopic Surgery (VATS) lobectomy for primary lung cancer and randomized to receive Low-Molecular-Weight Heparin (LMWH).

Anti–factor Xa (IU/mL)	LMWH group
**Perioperative**	
Median (95% CI)	0.00 (0.01–0.08)
**1.postoperative day**	
Mean (SD)	0.20 (0.14)
**2.postoperative day**	
Mean (SD)	0.15 (0.07)

Anti-factor Xa data are for blood-samples taken perioperative and on the 1. and 2.postoperative day.

Normally distributed data are shown as means (standard deviations), whereas non-normally distributed data are shown as medians and (95% confidence intervals).

Abbreviations: CI: Confidence intervals; N/n: Numbers; IU: International Unit; LMWH: Low-Molecular-Weight Heparin; mL: Milliliter; SD: Standard deviation.

## Discussion

In this randomized, controlled trial we found that in patients undergoing VATS lobectomy for primary lung cancer, the coagulation profile did not differ whether the patients were given LMWH or not. The effect of LMWH administered once daily thus seemed limited as estimated by a wide range of coagulation analyses. To our knowledge, the impact on coagulation of LMWH given to VATS lobectomy patients has not been investigated before. Furthermore, we did not find that patients with low-stage lung cancer were hypercoagulable preoperatively.

Due to the large amount of coagulation analyses performed, the risk of committing type I errors increases. Accordingly, we found no clear pattern in the three analyses that differed significantly between the LMWH and the no intervention group. We did not find that patients undergoing potential curative surgery for lung cancer were hypercoagulable preoperatively, except for FIBTEM MCF, which was elevated in approximately 45% of patients. This is in accordance with the findings by Attaran et al [5] and supports the assumption that the effect of LMWH on the coagulation system is limited. These patients suffer predominantly from early-stage lung cancer, and are not as likely in a hypercoagulable state as are patients with more advanced disease [5]. We used the widely applied regimen with dalteparin (Fragmin^®^) 5000 IE given once daily, and the lack of biochemical effect can probably be ascribed to the fact that the patients did not become hypercoagulable following surgery.

A lower level of Fibrin d-dimer in LMWH group on was found on the 1.postoperative day and 2.postoperative day, although not statistical significant. This, however, does not indicate the absence of an effect, but could because the study was underpowered.

The anti Factor Xa after prophylactic doses of LMWH is normally in the range of 0.2–0.5 IU/ml, which are measured 4 hours after injection [11]. However, blood-samples taken on the 1. and 2. postoperative day were obtained 10 hours after injection, and the preoperative samples were obtained approximately 12 hours after injection of LMWH. Accordingly, our results are lower than 0.2–0.5 IU/ml. Yet, the level of anti Factor Xa was elevated, documenting that the LMWH was administrated.

We did not analyse it in the preoperative blood-sample, since this was taken before the patients randomized to LMWH would receive LMWH. This analysis is only relevant in the group randomized to receive low-molecular-weight heparin (LMWH), since analysis of anti-Xa activity provides no information for patients not receiving LMWH or heparin.

The Prothrombin fragment 1 + 2 (PTF) and thrombin–antithrombin III complex (TAT) are generally considered as markers of thrombin generation [12].

Different types of coagulation analyses have been used to potentially predict VTE in cancer patients in several studies. Analyses covers thromboelastography [13], thrombin generation [14], markers of thrombin generation (prothrombin fragment 1 + 2) [15] and standard coagulation parameters [15]. The results of these coagulation analyses correlated with clinical events such as bleeding and VTE [16]. Attaran et al [5] recommended screening of patients to identify the patients being hypercoagulable and targeting thromboprophylaxis in this specific patient group. The question is how and when to perform such a screening; one approach could be to screen preoperatively using thromboelastography (TEG^®^ or ROTEM^®^). Future studies using clinical endpoints will hopefully clarify the potential value of this approach.

It should be emphasized that thrombin generation parameters obtained by calibrated automated thromboelastography estimate the thrombin generation potential in the patient, which not necessarily reflects activity *in vivo*. In order to reflect *in vivo* activity in the coagulation system and depict whether a patient is actively producing thrombin and/or fibrin, measurement of prothrombin fragment 1+2 or thrombin-antithrombin (TAT) should be used. In this study measurement of thrombin generation was used to describe the clotting potential and whether this was altered by LMWH treatment, and in order to depict active coagulation processes measurement of D-dimer was included.

Since we merely used surrogate endpoints, our study was mainly explorative and was not powered for detecting statistically differences regarding clinical endpoints. To gain further knowledge, a large study with clinical endpoints is needed.

However, in patients with low stage lung cancer undergoing VATS lobectomy, LMWH as prophylaxis administrated pre- and perioperatively had no significant impact on the coagulation system.

However, the potential effect of LMWH in reducing VTE could be a systematic effect and not reflected by the applied coagulation analysis, but as stated above there is a correlation between the analysis applied and the incidence of VTE [16].

Thoracic surgical clinical practice has over the recent years changed dramatically with the increasing use of minimal invasive surgery, which reduces the surgical trauma and hence facilitates recovery and mobilisation. We also demonstrated that VATS lobectomy only posed minimal impact on the coagulation system, which could be due to the minimal trauma using this approach [8].

The use of medical prophylaxis implies drawbacks as increased cost, an increased risk of bleeding, logistical difficulties, and pain due to the injection. However, more important than these drawbacks is that an optimized and more targeted and specific approach of thromboprophylaxis could be provided in the future. As stated in the introduction the recommendations regarding the use of LMWH is mostly based on observational studies.

Potentially, LMWH could be started after discharge from the hospital or merely given to those with a high stage tumour and/or given adjuvant chemotherapy. Alternatively, if other types of thromboprophylaxis (e.g. non-vitamin K antagonist oral anticoagulants) have a more profound impact on the coagulation analyses, they could potentially replace LMWH.

The strengths of the present study are the reliable study design in terms of randomization to different regimens, the multi-centre study design (providing a high external validity) and the use of advanced and validated coagulation analyses. Our study has of course limitations since we used surrogate and not clinical endpoints in terms of VTE and bleeding events. Accordingly, we found no difference in VTE, which our study was not designed or powered to detect (needs > 2000 patients in each group). From a methodological point of view, the results are limited by the fact that both ROTEM^®^ and trombin generation analyses reflect coagulation potential and not necessarily the *in vivo* coagulation activity.

On the positive side, we did not find any detrimental impact of LMWH either. We had a limited follow-up time of 30 days postoperatively. However, we find that this fully reflects the entire perioperative period. We had a relatively high dropout rate due to the complex set-up regarding blood sampling and analyses, and the potential interaction with medication (e.g. aspirin and NSAID). However, this should not interfere with either the internal and external validity of our study.

In conclusion, use of LMWH administered once daily as thromboprophylaxis did not alter the coagulation profile *per se*. As the present study primarily evaluated biochemical endpoints, further studies using clinical endpoints are needed in regards of an optimized thromboprophylaxis approach.

## Supporting information

S1 Table Dataset for patients. Data are presented for the Video-Assisted Thoracoscopic Surgery (VATS) low-molecular-weight heparin (LMWH) group and for the VATS none LMWH group, respectively.
(XLSX)

S2 Table Protocol (original) in Danish. (DOCX)

S3 Table CONSORT 2010 checklist. (DOC)

S4 Table Coagulation analysis. (XLSX)

S5 Table Protocol in English. (DOCX)
