# Supplementary material for: Coagulation profile in patients undergoing video-assisted thoracoscopic lobectomy: A randomized, controlled trial
Source: PLoS One. 2017 Feb 15;12(2):e0171809. doi: 10.1371/journal.pone.0171809 (PMC5310875; doi:10.1371/journal.pone.0171809)
Supplement: S2 Table — (DOCX) [file pone.0171809.s002.docx]

**Coagulation Profile in Patients undergoing Video Assisted Thorascopic Surgery (VATS) for lung cancer - A randomized, controlled trial**

**Koagulationsprofil hos patienter som opereres for lungekræft -**

**et randomiseret, kontrolleret studie**

Hjerte-, Lunge-, Karkirurgisk afdeling T

Klinisk Biokemisk afdeling

Anæstesiologisk-Intensiv afdeling I &

Klinisk Institut

Aarhus Universitetshospital

**Sponsor**

Århus den

……………………………………………………

Hjerte-, Lunge-, Karkirurgisk Afdeling T

Aarhus Universitetshospital Skejby

**Koordinerende investigator**

Århus den

……………………………………………………

Thomas Decker Christensen

Afdelingslæge, lektor, dr.med., ph.d.

Aarhus Universitetshospital

**Investigatorer**

Århus den

……………………………………………………

Søren Pedersen

Afdelingslæge

Aarhus Universitetshospital

Århus den

……………………………………………………

Anne-Mette Hvas

Overlæge, lektor, ph.d.

Aarhus Universitetshospital

Århus den

……………………………………………………

Henrik Vad

Afdelingslæge

Aarhus Universitetshospital

Århus den

…………………………………………………….

Henriette Bach Knudsen

Afdelingslæge

Aarhus Universitetshospital

**Baggrund**

Lungecancer er en hyppig og alvorlig sygdom med ca. 4200 nye tilfælde per år i Danmark, hvoraf ca. 25 % af patienterne bliver tilbudt operation [[1](#_ENREF_1)]. Der findes principielt to operationsmetoder: 1) åben operation via thorakotomi og 2) kikkertoperation (Video Assisted Thorascopic Surgery (VATS)). Sidstnævnte metode vinder mere udbredelse pga. et formodet mindre kirurgisk stress på patienten, hvilket bl.a. medfører færre smerter efter operationen [[2](#_ENREF_2)]. Hvorvidt VATS medfører en mindre aktivering af koagulationssystemet end åben operation vides ikke.

Generelt er patienter med cancer i en hyperkoagulabel tilstand pga. aktivering af koagulationssystemet, således at de har en øget risiko for f.eks. dyb venetrombose og lungeemboli [[3](#_ENREF_3)]. Denne hyperkoagulable tilstand skyldes bl.a., at tumor øger ekspressionen af prokoagulante faktorer som f.eks. tissue faktor og cancer procoagulant [[3](#_ENREF_3)].

Patienter med cancer, som undergår operative indgreb, påføres et operative stress, som også medfører aktivering af koagulationssystemet.

Hvorvidt man skal forsøge at modvirke denne tilstand med antitrombotisk medicin er uafklaret. Ofte bliver det anbefalet at give patienterne lavmolekylært heparin (LMWH) præ-, samt postoperativt. Patienter, som opereres for lungecancer på Aarhus Universitetshospital, får dagen før operation LMWH i form af inj. s.c. dalteparin (Fragmin®) 5000 IE x 1, som fortsættes postoperativt indtil udskrivelsen. Der foreligger dog ingen studier, som klart har belyst, hvorvidt det er nødvendigt at give patienterne antitrombotisk medicin, og i givet fald hvilken type. Da det endvidere er uvist, om denne profylakse er nødvendig, har man andre steder en anden procedure. Dette gælder f.eks. på Hjerte-, Lunge-, Karkirurgisk afdeling T, Odense Universitetshospital, hvor patienterne ikke som standard får forebyggende antitrombotisk medicin. Ved at give antitrombotisk medicin nedsættes risikoen potentielt for tromboembolier, men risikoen for blødning stiger. Der mangler viden og studier på området til at afklare forholdet mellem effekt og risiko.

Medicinen gives ud fra den antagelse, at når patienten undergår operation, påføres en yderligere forværring af deres hyperkoaguable tilstand, og risikoen for tromber og embolier øges. Attaran et al. [[4](#_ENREF_4)] fandt hos patienter, som gennemgik operation for lungecancer, at patienterne generelt ikke var hyperkoaguable. Dette kan dog skyldes, at der ikke blev anvendt metoder, som var følsomme nok til at registrere eventuelle ændringer i koagulationssystemet. Hvis man kan karakterisere den eventuelle hyperkoaguable tilstand, som patienter med lungecancer har, kan man potentielt modvirke denne tilstand mere specifikt rent medicinsk og dermed reducere disse patienters morbiditet peri- og postoperativt, og evt. forlænge deres levetid.

Endvidere kan man i senere studier afprøve forskellige former for antitrombotisk medicin og afklare hvilken type antitrombotisk medicin, patienterne vil have størst effekt af, bl.a. baseret på den karakteristik af koagulationsprofilen, som foretages i nærværende studie.

Ved at anvende nye analysemetoder i form af tromboelastometri (ROTEM^®^), trombingeneration og trombocytfunktionsundersøgelser kan den totale koagulationsprofil bedre karakteriseres, da man undersøger både trombocytfunktion og den globale hæmostatiske kapacitet [[3](#_ENREF_3), [5](#_ENREF_5)]. Man vurderer således ikke kun delkomponenter i koagulationen, men globale og dynamiske parametre.

Disse nye laboratorieanalyser korrelerer godt med det kliniske outcome [[6](#_ENREF_6)]. Således kan man anvende biokemiske effektparametre til potentielt at forudsige risiko for trombose og/eller blødning. Nærværende studie kan bibringe en ny og særdeles relevant viden om fundamentale elementer indenfor koagulation og lungecancer og dermed potentielt danne grundlag for et større studie med inddragelse af kliniske effektmål.

På baggrund af ovenstående er det relevant at undersøge, hvorvidt patienter med lungecancer er i hyperkoaguabel tilstand præ-, per-, og postoperativ samt undersøge om antitrombotisk medicin i form af LMWH kan modvirke denne tilstand.

**Titel**

Dansk: Koagulationsprofil hos patienter der opereres for lungekræft - et randomiseret, kontrolleret studie.

English: **Co**agulation **P**rofile in **P**atients undergoing **V**ideo **A**ssisted **T**horascopic **S**urgery (VATS) for lung cancer - A randomized, controlled trial.

Acronym: **COPPVATS**

**Hypoteser**

1. Patienter med lungecancer er i en hyperkoagulabel tilstand præ-, per-, og postoperativt
2. Patienter, som opereres for lungecancer er i en mere udtalt hyperkoagulabel tilstand per- og postoperativt sammenlignet med præoperativt
3. Patienter, som opereres for lungecancer, får deres hyperkoagulabilitet nedsat, når de får LMWH i profylaktisk dosering
4. At anvendelse af VATS bevirker en mindre hyperkoagulabel tilstand end åben operation hos patienter som opereres for lungecancer

**Formål**

1. At klarlægge patienter med lungecancers totale hæmostatiske/trombotiske kapacitet præ-, per- og postoperativt.
2. At undersøge om profylaktisk behandling med LMWH modvirker patienternes potentielle hyperkoagulabilitet
3. At undersøge om der er forskel på patienter, der får udført VATS eller åben operation mht. påvirkning af koagulationssystemet.

**Design:**

- Randomiseret, kontrolleret studie
- Prospektivt kohorte studie

**Endpoints**

Koagulabilitet målt vha. tromboelastometri (ROTEM^®^), trombingeneration, standard koagulationsanalyser samt trombocytfunktion.

**Inklusionskriterier**

- Skal opereres for cancer pulmonis med forventet lobektomi eller bilobektomi
- Skal have foretaget VATS operation (for de patienter som skal randomiseres til LMWH eller ingen profylakse)
- Skal være villig til at lade sig randomisere (gælder VATS-patienter)
- Skal være 18 år eller derover på indlæggelsesdagen
- Skal være i stand til at give informeret samtykke
- Kvinder skal anvende sikker antikonception i henhold til Lægemiddelstyrelsen retningslinier - se kommentar nedenfor^1^

**Eksklusionskriterier:**

- Tromboemboliske tilfælde indenfor de sidste 3 mdr. (både venøse og arterielle)
- Gravid
- Ammende
- Er i antikoagulerende behandling med en vitamin K-antagonist (f.eks. warfarin (Marevan®) eller direkte eller indirekte trombinhæmmer (dabigatran, apixaban eller rivaroxaban)
- Er i trombocythæmmende behandling med clopidogrel, magnyl, prasugrel eller ticagrelor, og der er ikke holdt pause minimum 5 dage før operationen (dog 7 dage ved prasugrel) med disse præparater.
- Overfølsomhed overfor LMWH

^1^ I forhold til inklusion af fertile kvinder, er det den enkelte investigators ansvar, i hvert enkelt tilfælde, at søge oplysninger hos kvinden om anvendelsen sikker antikonception, evt. menopause etc. Ved sikker antikonception forstås spiral eller hormonel antikonception (p-piller, implantat, transdermal depotplaster, vaginalring eller depotinjektion, anvendt i hele forsøgsperioden indtil kontrollen i ambulatoriet). Ved tvivl om mulig graviditet testes med u-stix.

**Randomisering**Alle henviste patienter, der skal opereres for lungekræft, og som umiddelbart efterlever inklusionskriterierne og ikke kan udelukkes i forhold til eksklusionskriterierne vil blive forespurgt om deltagelse i projektet.

VATS-patienterne vil blive randomiseret til enten:

1) Ingen tromboseprofylakse med LMWH eller

2) Tromboseprofylakse med dalteparin (Fragmin®) 5000 IE inj. s.c. * 1 dgl.

Der vil blive anvendt blok-randomisering (restricted randomisering) med varierende blokstørrelse for at få lige mange patienter i begge grupper. Hvis der deltager andre centre end Aarhus Universitetshospital, vil der blive stratificeret for dette i randomiseringen.

Randomiseringen foregår, efter at patienten er inkluderet (dagen før operationen), og det gennemføres via et webbaseret randomiseringsprogram, som er konstrueret af Advancer.dk.

**Blinding**

Patienterne og det personale, som giver medicinen, vil ikke være blindet, da der er tale om enten inj. s.c. eller ingen medikamina. Det skønnes ikke at være et problem, da det ikke har nogen betydning for bedømmelse af de anvendte effektmål (biokemiske analyser). Det personale, som analyserer blodprøverne, samt de som foretager dataanalysen, vil være blindet mht. hvilken intervention, som den pågældende patient har modtaget.

Blinding med inj. med placebo vil dog heller ikke give mening, da man som regel så tydeligt kan se hæmatomer ved stikmærkerne, når der er heparin i.

Hvis patienten får bivirkninger, der kan tilskrives projektmedicinen, eller får komplikationer i form af tromboembolier eller blødning, kontaktes en læge fra projektgruppen. Herefter planlægges den videre behandling bl.a. ud fra hvilken medicin, patienten har fået.

**Patientpopulation**

Konsekutive patienter, der indlægges med cancer pulmonis og som skal gennemgå operation for dette.

Da laboratorieanalyserne skal foretages af en forskningsbioanalytiker, er det kun muligt at få disse analyser foretaget på hverdage (mandag-fredag). Derfor vil der kun blive inkluderet patienter, som bliver opereret mandag, tirsdag eller onsdag. Der vil blive foretaget postoperative blodprøveanalyser til og med 2. postoperative dag.

**Data fra patienter som gennemgår en åben lobektomi**

Der planlægges at inkludere 30 patienter, som skal gennemgå åben lobektomi, men disse er ikke med i randomiseringsdelen. Alle patienter som gennemgår en åben operation får inj. s.c. Fragmin**®** 5000 IE *1 dgl. De inkluderede patienter matches med VATS patienterne, som er randomiseret til Fragmin**®**, og de matches på følgende parametre: Køn, alder samt cTNM^[[1]](#footnote-1)^.

Patienter, der gennemgår åben lobektomi, får foretaget samme blodprøveanalyser som de øvrige patienter. Alle åbne operationer foretages på Aarhus Universitetshospital.

**Data fra raske voksne**

Koagulabilitet bedømt vha. tromboelastometri (ROTEM^®^), standard koagulationsanalyser samt trombocytfunktion foreligger allerede hos raske voksne, og kan anvendes til datasammenligning (kontrol/baseline gruppe). Disse data er ved Klinisk Biokemisk Afdeling, Aarhus Universitetshospital.

**Deltagende centre**

På nuværende tidspunkt deltager ét center: Hjerte-, Lunge-, Karkirurgisk afdeling T, Aarhus Universitetshospital

Endvidere vil de thoraxkirurgiske centre på Aalborg Universitetshospital, Odense Universitetshospital samt Rigshospitalet blive adspurgt om deltagelse. Hvis disse centre giver tilsagn, laves en tillægsprotokol vedrørende dette.

**Dataopsamling/ kildedata**

Antal patienter i hele populationen (alle patienter indlagt med cancer pulmonis og som skal opereres for dette på hjerte-lunge-karkirurgisk afdeling T, Aarhus Universitetshospital, i inklusionsperioden)

Indlæggelse data/baseline variable på alle inkluderede patienter:

- Fødselsdato
- Køn
- Vægt
- Højde
- Rygestatus
- Øvrige sygdomme (inkl. misbrug)
- Medicin
- Peroperative og postoperative data/baseline variable: Side (hø./ve.), lokalisation (over-, mellem- eller underlap), (cTNM), makro- og mikroskopisk radikalitet, thorakotomi (anterolateral/posterolateral), intraperikardiel dissektion, tumor størrelse, mikroskopidiagnose (planocellulært, adenocacinom etc.), patologisk staging (pTNM)
- Blødning i ml. per-, postoperativt. Målt som det blod, som kommer i suget under operationen, postoperativt som det, som kommer i drænet efter operationen (bliver registreret i PDM eller journalen)
- Operationsdata (tid, komplikationer, medicamina givet samt anæstesiologiske og hæmodynamiske parametre)
- Forløb under indlæggelse ved udskrivelse/overflytning (epikrise)
- Forløb og medicin givet af evt. modtagende sygehus (epikrise rekvireres)
- Indlæggelsestid på evt. modtagende sygehus (epikrise rekvireres)
- Opfølgning dag 14 i ambulatoriet på Hjerte-, Lunge-, Karkirurgisk afd.T mht. medicin og forløb siden udskrivelsen
- Død, cerebral apopleksi/TCI og AMI efter dag 30 (Gøres via telefonisk kontakt til patienterne, egen læge samt hjemstedsygehus).

**Laboratorieanalyser**

Der tages prøver på følgende tidspunkter:

1. Præoperativt om morgenen på operationsdagen^[[2]](#footnote-2)^ (før patienten får dalteparin (Fragmin®) 5000 IE inj. s.c. * 1.
2. Perioperativt: Når lappen er taget ud (dvs. når bronkus er staplet).
3. Postoperativt:
   1. Kl. 12.00 1. dag efter operationen.
   2. Kl. 12.00 2. dag efter operationen.

Alle prøverne tages på Hjerte-, Lunge-, Karkirurgisk afdeling T (afsnit T3) eller på opvågningsafsnittet. Blodprøverne tages af en forskningsbioanalytiker med anvendelse af blodprøveglas, jf. nedenfor. Herefter fragtes blodprøven til Klinisk Biokemisk Afdeling. Analyserne på ROTEM^®^ og trombocytfunktionsundersøgelser foretages ½ time og højst 2 timer efter blodprøvetagningen. Resten af blodprøverne analyseres i rutinen på Klinisk Biokemisk Afdeling eller centrifugeres, afpipetteres og fryses ved – 80^o^C.

Ved baseline målinger (præoperativt) foretages følgende sikkerhedsanalyser:

B- Hæmoglobin, B - Leukocytter, B – Trombocytter, B – Hæmatokrit, INR, P - CRP; massek., P - Fibrinogen; massek., faktor VIII:clot, Fibrin D-dimer.

Præoperativt, perioperativt og postoperativt på ovennævnte tidspunkter foretages:

- - Tromboelastometri (ROTEM^®^) med analyserne in-tem, ex-tem, fib-tem og hep-tem. Der registreres følgende parametre
    - Clotting time (CT (sec)), propagation (MaxVel (mm*100/sec) t,MaxVel, s) og termination (maximum clot firmness (MCF)(mm*100/sec)
  - Trombingeneration: 1) recalcificering, 2) aktivering med tissue factor 1:17,000.
    - Parametre:
      - Lag time [min], ETP [nM thrombin*minute], peak levels of thrombin generation [nM thrombin] og time to peak thrombin (ttpeak) [min].
  - Standard koagulationsanalyser: APTT, INR, fibrinogen, fibrin d-dimer, trombintid, trombocyttal. Derudover analyseres faktor VIII: Clot
  - Trombocytfunktion, taget i 3 ml hirudinglas og analyseres på Multiplate^®^ med anvendelse af agonisterne ADP 6,4 µM (ADP-test), Collagen 3.2 µg/mL (COL-test) og TRAPtest. Trombocytaggregationen udtrykkes ved Aggregation unit (AU), area under curve (A*min).

Volumen blod der tages i alt pr patient:

Pr. Rotem: 3,6 ml citratplasma

Pr. Multiplate: 3 ml hirudin (i alt for alle 3 agonister)

Pr. Trombingeneration: 3,6 ml citratplasma

Hæmglobin, leucyttal, trombocyttal, hæmatokrit: 3 ml EDTA

CRP: 3 ml litium-heparin glas

APTT, trombintid, fibrinogen, fibrin d-dimer, INR og faktor VIII.Clot: 1,8 ml citratplasma.

Maksimal total volumen af alle projekt blodprøverne pr. patient: 58,8 ml.

Deltagerne vil desuden blive spurgt om de vil afgive et ekstra glas blod (4 ml) til opbevaring i en biobank. Biobanken er på Klinisk Biokemisk Afdeling, Aarhus universitetshospital, og prøven gemmes med henblik på fremtidig forskning. Forsøgspersoner kan godt deltage i projektet uden at afgive blodprøve til biobanken. Biobankens materiale vil kun blive anvendt i fremtidige projekter efter godkendelse af Videnskabsetisk Komité og efter aftale mellem projektsamarbejdspartnerne. I tilfælde af at en forsøgsdeltager henvender sig med ønske om at få fjernet sit blod fra biobanken, vil dette blive imødekommet, men allerede producerede resultater vil ikke blive slettet. Materialet opbevares i biobanken 15 år efter patientinklusionens afslutning.

**Håndtering og arkivering af data**

Alle data føres i en Case Report Form og overføres til et database-program med henblik på statistisk bearbejdning/ analysering ved hjælp af dobbeltindtastning. Ved håndtering, bearbejdning og arkivering af indsamlede data følges Datatilsynets retningslinjer, hvilket bl.a. indebærer, at alle personhenførbare data slettes ved projektets afslutning. Indsamlede data opbevares på Hjerte-, Lunge-, Karkirurgisk afd.T, Aarhus Universitetshospital.

**Projektmedicin**

Dalteparin (Fragmin®): 5000 IE s.c. * 1 dgl.

I den gruppe, som randomiseres til dalteparin (Fragmin®) 5000 IE s.c. * 1, gives dette ca. kl. 1400 dagen før operationen^[[3]](#footnote-3)^ (ca. 20 timer før operationen), og denne medicin fortsætter patienten med indtil udskrivelse, hvor behandlingen seponeres.

Der anvendes Dalteparin (Fragmin®) 5000 IE s.c. fra afdelingens egen beholdning (hyldemedicin). Projektmedicinen håndteres og indgives af sygeplejersker på afdeling T, som under indlæggelsen og i forsøgsperioden er patienternes stamafdeling. Sygeplejerskerne er vant til at håndtere Dalteparin (Fragmin®) s.c.

Der føres medicinregnskab med forsøgsmedicinen på patientniveau på et særskilt dokument.

**Øvrig medicin**

Patienten fortsætter med sin vanlige medicinske behandling helt op til operationen, fraset clopidogrel og/eller magnyl, prasugrel samt ticagrelor, som pauseres 5 dage (dog 7 dage ved prasugrel) før operationen, hvilket er vanlig procedure på afdelingen. Denne medicin genoptager patienten på 3. postoperative dag.

Patienten genoptager sin øvrige vanlige medicin fra 1.postoperative dag.

**Interaktion**

Samtidig indgift af farmaka med hæmmende virkning på hæmostasen, fx acetylsalicylsyre og andre NSAID, vitamin K-antagonister og dextran, kan forstærke den antikoagulerende virkning. Dette er dog ingen relativ eller absolut kontraindikation. Pga. interaktion med de analyser, som udføres (trombocytfunktionsundersøgelse), så må disse medikamenter ikke gives til patienterne de første to postoperative dage.

**Intervention**

Patienter som indlægges til VATS-operation for ovenstående, opfylder in-, og exclusionskriterierne, og er villige til at deltage randomiseres til følgende grupper:

1. Ingen tromboseprofylakse
2. Tromboseprofylakse med dalteparin (Fragmin®) 5000 IE inj. s.c. * 1 dgl.

Efter s.c. inj. med LMWH er der peak værdi efter 4 timer, 50% af peak værdi efter 12 timer. Patienter får før operationen LMWH om eftermiddagen, i operationsdøgnet ingen LMWH, og fra og med 1.postoperative dag, gives det kl. 0800 indtil udskrivelsen.

**Styrkeberegning**

Studiet er primært et eksplorativt/hypotesegenerende studie, og styrkeberegningen er derfor behæftet med nogen usikkerhed. Vi har baseret vores styrkeberegning på tromboelastometri (ROTEM) ud fra følgende antagelser: Hovedeffektmålet er forskel i CT-værdien på analysen EX-TEM mellem de to grupper VATS-patienter, der er randomiseret til hhv. LMWH eller ingen intervention. På baggrund af etablering af referenceinterval ved vi, at gennemsnittet hos voksne er 60 sec og standard deviationen er 25 sec. Når vi antager en mindste relevante differens (MIREDIF) på 20 sec, et signifikans niveau på 5% (2α) og en styrke på 90% (1-β) skal der være 27 i hver gruppe. For at tage højde for missing values påregner vi at inkludere 30 patienter i hver gruppe. I alt 60 patienter, der skal have foretaget VATS lobektomi. Derudover inkluderes 30 patienter, som skal have foretaget åben kirurgi.

**Risici ved deltagelse og etiske overvejelser**

| Almindelige (1-10%) | Reaktioner og ubehag på indstiksstedet.  Forhøjede levertransaminaser.  Blødningstendens, trombocytopeni (type I).  Alopeci. |
| --- | --- |
| Ikke almindelige (0,1-1%) | Blødning (intrakraniel).  Trombocytopeni (type II).  Allergiske reaktioner. |
| Meget sjældne (under 0,01%) | Hæmatom ved epidural eller spinal punktur.  Hudnekrose. |

Bivirkningerne i forbindelse med kort tids brug af dalteparin (Fragmin®) 5000 IE inj. s.c. * 1 dgl. er få. Der er i litteraturen beskrevet komplikationer i form af (taget fra www.medicin.dk):





Der er set anafylaktisk reaktioner og retroperitoneal blødning. Frekvens ukendt.

Almindelig klinisk praksis er d.d. meget divergerende; Standardbehandlingen på Aarhus Universitetshospital er, at patienterne får tromboprofylaktisk behandling med LMWH, hvorimod patienter, der opereres for lungecancer på Odense Universitetshospital, ikke får lignende profylakse. Der er ingen sikker evidens for nogen af regimerne, hverken nationalt eller internationalt.

Derudover er der som ved enhver blodprøvetagning altid en lille risiko for infektion ved indstiksstedet og risiko for mindre hæmatomdannelse. Patienterne får i forbindelse med deltagelse taget maksimalt 58,8 ml ekstra blod, hvilket ikke vurderes at udgøre en risiko. Den enkelte patient vil ikke få gavn af at deltage. Resultaterne af studiet vil primært komme fremtidens patienter til gode. Samlet vurderer vi, at den viden, der opnås ved projektet, opvejer den ulempe der må være for patienterne ved at deltage

Projektet gennemføres i overensstemmelse med Helsinki II deklarationen. De Videnskabsetiske komitéer for Region Midtjylland, Datatilsynet samt Lægemiddelstyrelsen ansøges om tilladelse til at gennemføre studiet. Projektet vil følge retningslinjerne for Good Clinical Practice (GCP) og vil blive monitoreret via GCP-enheden, Aarhus Universitetshospital.

Oplysninger om forsøgspersonerne beskyttes efter Lov om behandling af personoplysninger og Sundhedsloven, og forsøget er anmeldt til Datatilsynet/Region Midtjylland.

**Afbrydelse af forsøget**

Ved mistanke om alvorlige bivirkninger i forbindelse med medicinen (Suspected Unexpected Severe Adverse Reactions (SUSAR)) afbrydes forsøget for den enkelte patient.

**Tidligere ophør af forsøget for den enkelte patient**

- Hvis patienten ønsker at forlade studiet.
- Hvis der opstår blødnings- eller tromboemboliske komplikationer
- Hvis der opstår bivirkninger til medicinen

Ved afbrydelse vil patienten følge afdelingens vanlige procedure for behandling af patienter, som opereres for cancer pulmonis.

**Hændelser og bivirkninger**

Alle utilsigtede hændelser og bivirkninger i hele behandlingsperioden og indtil sidste samtale efter 30 dage registreres. Alle Adverse Events registreres i patienternes Case Report Form.

Alle Serious Adverse Events skal rapporteres af investigator til sponsor indenfor 24 timer efter at investigator har fået kendskab til den alvorlige hændelse. Ved Serious Adverse Events forstås som en hændelse eller bivirkning, som resulterer i død, er livstruende, medfører hospitalsindlæggelse eller forlængelse af hospitalsophold, resulterer i betydelig eller vedvarende invaliditet eller uarbejdsdygtighed.

Alle SAE´s skal følges indtil problemet er løst eller indtil det er afgjort, at deltagelse i forsøget ikke var årsagen.

SUSAR, som er dødelige eller livstruende, indføres i registreringsskemaet (Indberetning af SAE/SUSAR), og vil indenfor 7 dage blive indberettet til lægemiddelstyrelsen og De Videnskabsetiske komitéer for Region Midtjylland, jf. ” Vejledning til indberetning af mistænkte uventede og alvorlige bivirkninger set i kliniske forsøg” fra medicinopstart til ambulant kontrol (ca. postoperative dag 20).

Alle andre SUSAR skal indberettes til Lægemiddelstyrelsen senest 15 dage efter, at sponsor har fået kendskab til disse. Indberetningen foregår vha. ”Indberetning af SAE/SUSAR”, bilag 4.

**Information til patienter ved indhentning af informeret samtykke**

Patienter, der kan inkluderes i projektet, får ved indlæggelsen både skriftlig og mundtlig information i uforstyrrede fysiske rammer. Denne information foretages af en projektsygeplejerske, som er særligt instrueret i dette. Patienterne får mindst 24 timers betænkningstid inden indhentelse af samtykket, og patienten får ved første samtale af vide, at vedkommende er velkommen til at medbringe en bisidder ved den efterfølgende samtale. Den informerende projektsygeplejerske understreger under samtalen, at det er frivilligt at deltage i projektet. Hvis patienten ikke ønsker at deltage i projektet, følger patienten den vanlige praksis i forbindelse med behandling af cancer pulmonis. Patienten kan selvfølgelig trække sig ud af projektet på et hvilket som helst tidspunkt, uden at det vil påvirke patientens nuværende eller fremtidige behandling.

Patienterne får endvidere ved informationen udleveret: ”Forsøgspersoners rettigheder i et sundhedsvidenskabeligt forskningsprojekt”.

Den enkelte patient vil blive informeret såvel mundtligt som skriftligt af en projektsygeplejerske samt af en person fra projektgruppen efter at være blevet oplyst om deres ret til at medbringe en bisidder ved den mundtlige information.

Der skal afgives mundtligt og skriftligt informeret samtykke, inden vedkommende kan inkluderes i studiet.

**Dataanalyse**

Microsoft® Office Excel 2003 med Analysis-it™ version 1.71 (Analysis-it Software Ltd.) anvendes til den statistiske analyse.

Endpoints vil for den enkelte gruppe blive beskrevet med enten middel og standarddeviation eller median og percentil, afhængigt af om data er normalfordelte eller ej. De to VATS-gruppers endpoints sammenlignes med enten Student's uparret t-test eller en Mann-Whitney U-test afhængigt af, om det er rimeligt at antage, at variationen mellem grupperne er normalfordelt.

Alle data analyseres efter intention to treat princippet. Signifikansniveauet er sat til 0,05. Sammenligning vil ske mellem VATS-grupperne mht. præ-, per-, og postoperative data. Derudover sammenlignes præ-, per-, og postoperative data for den VATS-gruppe som fik LMWH med patienter, der gennemgik åben kirurgi. Data fra raske voksne (som allerede haves) vil blive sammenlignet med præoperative data fra projektdeltagerne.

**Aftaler med andre afdelinger/sygehuse**

- Advancer.dk vedr. konstruktion af database samt udarbejdelse af randomiseringsmodul samt database.
- GCP-enheden (Good Clinical Practice) ved Aarhus Universitetshospital vedr. monitorering af studiet.
- Klinisk Biokemisk Afdeling, Aarhus Universitetshospital vedr. udtagning samt analyse af blodprøver.
- Anæstesiologisk-Intensiv Afdeling på Skejby vedr. registrering af data peri- samt i den umiddelbare postoperative periode.

De thoraxkirurgiske centre på Aalborg Universitetshospital, Odense Universitetshospital samt Rigshospitalet vil blive adspurgt om deltagelse.

**Anmeldelser**

- Videnskabsetisk komite
- Lægemiddelstyrelse (Sundhedsstyrelsen)
- Datatilsynet/Region Midtjylland
- [www.clinicaltrials.gov](http://www.clinicaltrials.gov)

**Økonomisk støtte**

Projektet er ikke støttet af kommercielle økonomiske midler. Offentlige og private fonde vil blive ansøgt om økonomisk støtte.

Ingen projektdeltagere har personligt økonomisk incitament til at gennemføre projektet.

**Forsikring**

Deltagerne er dækket i overensstemmelse med patientforsikringsloven samt lov om erstatning for lægemiddelskader. Det undersøgende personale er dækket af ansvars- og arbejdsskadeforsik-ringen tegnet af Aarhus Universitetshospital.

**Afslutning af forsøget**

- For hver enkelt patient vil der ved afslutning af studiet blive udfærdiget en Case Report Form.
- Lægemiddelstyrelsen underrettes senest 90 dage efter forsøgets afslutning og snarest muligt og senest inden 1 år herefter indsendes forsøgets resultat til styrelsen, jf. lov om lægemidler § 89, stk. 2, nr. 4.
- De Videnskabsetiske komitéer for Region Midtjylland orienteres vha. afsluttende forskningsrapport eller publikation der sendes til komiteen.

**Tidsplan**

Selve studiet: Første patient planlægges inkluderet i August 2012, og sidste patient forventes inkluderet i august 2014. Da den sidste patient skal følges i 30 dage efter operationen, forventes projektet afsluttet september 2014.

Tidsplanen afhænger af, hvor mange centre, der ønsker at deltage. På de enkelte centre udføres der per år ca. følgende antal VATS-lobektomier:

- Aarhus Universitetshospital: 50
- Odense Universitetshospital: 75
- Rigshospitalet: 125
- Aalborg Sygehus 10

Der bliver foretaget minimum dobbelt så mange åbne lobektomier som VATS lobektomier.

**Budget**

Se bilag 1

**Bilag**

1. Budget
2. Samtykkeerklæring
3. Patientinformation
4. Indberetning af SAE/SUSAR

**Publikationer**

Såvel positive,negative som inkonklusive resultater fra studiet vil blive publiceret i internationale tidsskrifter. Forskernes frie ret til publikation kan ikke begrænses. Alle oplysninger vil blive videregivet i anonymiseret form. Resultaterne vil desuden blive præsenteret ved internationale videnskabelige kongresser.

1. S Pedersen, H Vad, HB Knudsen, HK Pilegaard, AM Hvas, TD Christensen. Coagulation profile in patients undergoing lobectomy due to lungcancer
2. S Pedersen, H Vad, HB Knudsen, HK Pilegaard, AM Hvas, TD Christensen. The effect of low molecular heparin in patients undergoing lobectomy due to lungcancer - A Randomized, Controlled Trial.
3. S Pedersen, H Vad, HB Knudsen, HK Pilegaard, AM Hvas, TD Christensen. Difference in coagulation profile between VATS and open lobectomies - A prospective cohort study
4. AM Hvas, S Pedersen, H Vad, HB Knudsen, HK Pilegaard, TD Christensen. Platelet function in patients undergoing lobectomy due to lungcancer

**Stedet for forsøgets udførelse**

Hjerte-, Lunge-, Karkirurgisk afdeling T

Aarhus Universitetshospital

Brendstrupgaardsvej 100

8200 Aarhus N

**Projektdeltagere samt ansvarsfordeling**

Projektleder fra afdeling T, Aarhus Universitetshospital: Thomas Decker Christensen

Klinisk ansvarlig læge ved afdeling T, Aarhus Universitetshospital: Hans K. Pilegaard

Praktisk projektleder: Søren Pedersen, Anæstesiologisk-Intensiv afdeling I, Aarhus Universitetshospital

**Projektgruppe**

Thomas Decker Christensen. Afdelingslæge, lektor, dr.med., ph.d.

Hjerte-Lunge-Karkirurgisk afdeling T og Klinisk Institut, Aarhus Universitetshospital.

Hovedansvarlig for planlægning og design af studiet, praktiske gennemførelse af studiet, dataanalyse og endelig godkendelse af manuskript. Koordinerende investigator.

Hans K. Pilegaard, forskningsoverlæge, lektor

Hjerte-Lunge-Karkirurgisk afdeling T og Klinisk Institut, Aarhus Universitetshospital.

Klinisk og forskningsansvarlig. Medvirker til planlægning og design af studiet, den praktiske gennemførelse af studiet, dataanalyse og manuskriptudfærdigelse. Investigator.

Henrik Vad. Afdelingslæge

Hjerte-Lunge-Karkirurgisk afdeling T og Klinisk Institut, Aarhus Universitetshospital.

Medvirker til planlægning og design af studiet, den praktiske gennemførelse af studiet, dataanalyse og manuskriptudfærdigelse. Investigator.

Søren Pedersen. Afdelingslæge

Anæstesiologisk-Intensiv afdeling I, Aarhus Universitetshospital.

Medvirker til planlægning og design af studiet. Ansvarlig for den praktiske gennemførelse af studiet, dataanalyse samt første udkast til manuskript. Investigator.

Henriette Bach Knudsen, Afdelingslæge

Anæstesiologisk-Intensiv afdeling I

Medvirker til den praktiske gennemførelse af studiet, dataanalyse og manuskriptudfærdigelse. Investigator.

Anne-Mette Hvas. Overlæge, lektor, ph.d.

Klinisk Biokemisk Afdeling og Klinisk Institut, Aarhus Universitetshospital.

Medvirker til planlægning og design af studiet, den praktiske gennemførelse af studiet, analyse af blodprøver, dataanalyse og manuskriptudfærdigelse. Investigator

Vibeke Lauersen. Projektsygeplejerske.

Hjerte-lunge-karkirurgisk afd.T, Århus Universitetshospital, Skejby.

Praktisk gennemførelse af studiet.

**Referencer**

1. Jakobsen, E., et al., *Data from a national lung cancer registry contributes to improve outcome and quality of surgery: Danish results.* Eur.J.Cardiothorac.Surg., 2009. 35(2): p. 348-352.

2. Rueth, N.M. and R.S. Andrade, *Is VATS lobectomy better: perioperatively, biologically and oncologically?* Ann Thorac Surg, 2010. 89(6): p. S2107-11.

3. Papa, M.L., et al., *Thromboelastographic profiles as a tool for thrombotic risk in digestive tract cancer.* Exp.Oncol., 2007. 29(2): p. 111-115.

4. Attaran, S., P. Somov, and W.I. Awad, *Randomised high- and low-dose heparin prophylaxis in patients undergoing thoracotomy for benign and malignant disease: effect on thrombo-elastography.* Eur.J.Cardiothorac.Surg., 2010. 37(6): p. 1384-1390.

5. Sorensen, B., et al., *Whole blood coagulation thrombelastographic profiles employing minimal tissue factor activation.* J.Thromb.Haemost., 2003. 1(3): p. 551-558.

6. Fenger-Eriksen, C., et al., *Fibrinogen substitution improves whole blood clot firmness after dilution with hydroxyethyl starch in bleeding patients undergoing radical cystectomy: a randomized, placebo-controlled clinical trial.* J.Thromb.Haemost., 2009. 7(5): p. 795-802.

1. Dette er den kliniske (præoperative) stadieindeling af canceren ud fra størrelse af tumor, lymfeknuder samt metastaser (**T**umor, **N**odula, **M**etastasis) [↑](#footnote-ref-1)
2. For de patienter, som skal opereres om mandagen, tages denne blodprøven om fredagen (inden weekenden) [↑](#footnote-ref-2)
3. For de patienter som skal opereres om mandagen, gives det således om søndagen [↑](#footnote-ref-3)
