# Supplementary material for: Coagulation profile in patients undergoing video-assisted thoracoscopic lobectomy: A randomized, controlled trial
Source: PLoS One. 2017 Feb 15;12(2):e0171809. doi: 10.1371/journal.pone.0171809 (PMC5310875; doi:10.1371/journal.pone.0171809)
Supplement: S5 Table — (DOCX) [file pone.0171809.s005.docx]

**PONE-D-16-37887**

**Coagulation profile in patients undergoing video-assisted thoracoscopic lobectomy: a randomized, controlled trial**

**Thomas Decker Christensen et al**

**English protocol**

There has not been made any deviations from this study protocol in the Methods section of the manuscript.

In the original protocol in Danish, there is an arm (cohort study) of patients undergoing open locetomies, where all patients had low-molecular weight heparin prescribed. This part of the study is not mentioned thoroughly in this protocol, since these results will be published in another paper.

**Title:**

Danish: Koagulationsprofil hos patienter der opereres for lungekræft - et randomiseret, kontrolleret studie.

English: **Co**agulation **P**rofile in **P**atients undergoing **V**ideo **A**ssisted **T**horascopic **S**urgery (VATS) for lung cancer - A randomized, controlled trial.

Acronym: **COPPVATS**

**Background:**

Lung cancer is a serious and very frequent disease. For those 25% of the patients who will undergo surgery, there are two different methods of operation: either open surgery (thoracotomy) or Video Assisted Thorascopic Surgery (VATS). The latter method is increasing rapidly in use.

Patients are often prescribed low-molecular weight heparin (LMWH) given once daily pre- and postoperatively, but it is unclear whether this prophylactic treatment is necessary

Furthermore, it is unclear whether the coagulation system changes pre, per- and postoperatively in patients undergoing surgery for lung-cancer, and hereby it is unclear whether these patients need some sort and/or type of medical prophylactic treatment.

By using new laboratory methods in terms of thromboelastometry, thrombin generation and platelet function analyses, the entire coagulation profile can be characterized and hereby potentially provides more knowledge regarding the risk of thrombosis and bleeding during and after surgery.

**The purposes of the study are:**

- To estimate total haemostatic / thrombotic capacity pre, per-and postoperatively in patients with lung cancer who will undergo surgery.
- To investigate whether prophylactic treatment with Low Molecular Weight Heparin (LMWH) affects the patient's potential hypercoagulability.
- To investigate whether there are differences in patients who will undergo Video Assisted Thorascopic Surgery (VATS) or open surgery in regards to impact on the coagulation system.

**Eligibility:**

Inclusion Criteria:

- Undergo surgery for lung cancer with an expected lobectomy or bilobectomy
- The surgery shall be VATS
- Willing to be randomized (VATS-patients)
- Over 18 years old.
- Able to give assigned informed consent
- Women should be prescribed secure anticonception.

Exclusion Criteria:

- Thromboembolic events within the last three months (both venous and arterial)
- Pregnant
- Lactating
- Treatment with anticoagulation therapy in terms of vitamin K antagonist or direct or indirect thrombin inhibitors (dabigatran, apixaban or rivaroxaban)
- Treatment with platelet inhibitors in terms of Clopidogrel, ASA (acetylsalicylic acid), prasugrel and ticagrelor, and no pause of minimum 5 days (7 days for prasugrel) before surgery
- Allergy for LMWH

The patients will be included after oral and written consent.

The protocol for the study will complied with the Helsinki II declaration and shall be approved by the local scientific ethical committee, The Danish Data Protection Agency and by The Danish Board of Health.

The study will comply according to Good Clinical Practice (GCP) standards and monitored and approved by the GCP-unit, Aarhus University Hospital, Aarhus, Denmark. The trial is to be registered at ClinicalTrials.gov.

Serious adverse events (SAE) and Suspected Unexpected Serious Adverse Reactions (SUSAR) will be reported to The Danish Board of Health.

**Conditions and design:**

| [**Condition**](https://clinicaltrials.gov/ct2/help/conditions_desc) | [**Intervention**](https://clinicaltrials.gov/ct2/help/interventions_desc) | [**Phase**](https://clinicaltrials.gov/ct2/help/phase_desc) |
| --- | --- | --- |
| Lung Cancer | Drug: Dalteparin (Fragmin®) | Phase 4 |

| Study Type: | Interventional |
| --- | --- |
| Study Design: | Allocation: Randomized  Endpoint Classification: Safety/Efficacy Study  Intervention Model: Parallel Assignment  Masking: Single Blind (Subject)  Primary Purpose: Treatment |
| Official Title: | Coagulation Profile in Patients Undergoing Video Assisted Thorascopic Surgery  (VATS) for Lung Cancer - A Randomized, Controlled Trial |

**Arms:**

Experimental: Dalteparin (Fragmin®)

Dalteparin (Fragmin®) 5000 IU (International Unit) once daily

No Intervention: No treatment

No treatment

Open surgery arm:

Dalteparin (Fragmin®) 5000 IU once daily

**Randomization and blinding:**

Patients are randomly assigned to LMWH or no intervention/medication using a computerized prospective randomization schedule. Randomization is performed in blocks with various sizes in numbers of 2, 4 and 6 without blinding of allocation.

**Outcomes:**

Primary Outcome Measures:

- Thromboelastometry (ROTEM®): Analysis of in-tem, ex-tem, fib-tem og hep-tem and measurement of: Clotting time (CT (sec)), propagation (MaxVel (mm*100/sec) t,MaxVel, s) og termination (maximum clot firmness (MCF)(mm*100/sec)

Secondary Outcome Measures:

- Thrombin generation: 1) recalcificering, 2) activation with tissue factor 1:17,000 and estimation of Lag time [min], ETP (endogenous thrombin potential) [nM (nanomole) thrombin*minute], peak levels of thrombin generation [nM thrombin] and time to peak thrombin (ttpeak) [min].

Other Outcome Measures:

- Standard coagulation analysis: APTT (activated partial thromboplastin time), INR (International Normalized Ratio), fibrinogen, fibrin d-dimer, thrombin time, thrombocytes and Factor VIII: clot.
- Platelet function analysis: Analysis on Multiplate®Analyzer, where the platelet aggregation is expressed as area under the curve (AUC, AU*min).

##### **Observation period:**

It is not possible to perform the coagulation analyses in weekends, so only patients operated on Mondays, Tuesdays and Wednesdays are included.

All patients will be contacted by phone 30 days after the operation and systematically asked about complications, especially regarding VTE and bleeding events. At that time they will be terminated from the study.

**Termination of the study:**

Normally 30 days after inclusion.

Before if the patients wish to leave the study, bleeding- or thromboembolic complication, side-effects of the medication (LMWH), SAE or SUSAR

**Baseline characteristics and data:**

This will age, gender, co-morbidity, staging of the cancer, surgery performed, amount of blood/fluid in the drains, complications etc. (has been thoroughly defined in the Clinical Report File (CRF)).

Data will be obtained from patient records, information from the patients and the results of the analysis of blood-samples.

**Laboratory analyses:**

Blood samples are obtained and analysed at the following four time-points: 1) Preoperatively; the day before surgery (and before LMWH potentially are given); 2) Perioperatively at the time of stapling the bronchus; 3) Postoperatively 0800 AM at day 1; and 4) Postoperatively 0800 AM at day 2. The first 2 ml of blood are discarded before drawing blood into tubes containing sodium citrate for ROTEM^®^ analyses, thrombin generation and standard coagulation analyses including: Activated partial thromboplastin time (APTT), International Normalized Ratio (INR), fibrinogen (functional), fibrin d-dimer, thrombin time, platelet count and factor (F) VIII:Clot. Blood for ROTEM^®^ analyses are left at room temperature for 30 minutes before processing, whereas remaining analyses are done either as routine analyses or blood samples are centrifuged at 2800 g for 25 minutes and plasma are stored in aliquots at - 80 °C.

Regarding thromboelastometry (ROTEM^®^, Tem International GmbH, Munich, Germany), three standard assays are performed: INTEM, EXTEM, and FIBTEM. We obtained the dynamic parameters of clot initiation (clotting time: CT, seconds (s)) and clot propagation (maximum velocity of clot formation: MaxVel, mm x 100/s, time to maximum velocity: tMaxVel, s). Whole blood clot strength are assessed by maximum clot firmness (MCF, mm x 100).

Thrombin generation are evaluated by calibrated automated thrombogram (CAT; Thrombinoscope BV, Maastricht, the Netherlands) using platelet-poor plasma. The following parameters are analysed: Lag-time until initial thrombin generation (minutes), maximum concentration of thrombin (peak, nM), time to peak (ttpeak, minutes), and the endogenous thrombin potential (ETP, nM x minutes).

Reference values for the ROTEM^®^ are calculated based on data obtained from 73 healthy individuals previously published, while reference values for thrombin generation are obtained from 32 individuals published by Collins PW et al.

Preoperatively, the following baseline analyses are performed: haemoglobin, leukocyte andplatelet counts, creatinine, INR and C-reactive protein (CRP).

Preoperative (baseline) data in terms of clinical characteristics are collected systematically from medical records. Furthermore, peri- and postoperative data (operating time, bleeding during surgery, total drain loss, VTE and adverse events, length of stay and pathological staging) are registered prospectively in the case report form.

**Statistical analyses and sample size:**

Baseline data, peri- and postoperative characteristics are presented using descriptive statistics. Results of the coagulation analyses are tested for normal distribution and hence presented as either mean and standard deviation (SD) or median and 95% confidence interval (CI) or as minimum to maximum values. Normally distributed data are compared using Student’s unpaired t-tests, while non-normally distributed data are compared using Mann-Whitney U-tests.

Microsoft® Excel® for Mac 2011 (Microsoft®, Seattle, USA) and GraphPad Prism for Mac (GraphPad Software, Inc., CA, USA) are used for the statistical analyses.

The study is primarily an explorative study, and the samples size is therefore associated with some uncertainty. We based it on the EXTEM: CT from tromboelastometry. Normally the mean is 60 sec with a SD of 25 sec. The minimal relevant difference is estimated to be 20 sec. In order to detect this difference with a type I error of 0.05 and 90% power, 27 patients are needed in each group. Due to the anticipated failure of some patients in terms e.g. missing values, 30 patients in each group are considered appropriate.

Analysis is done using the intention to treat principal.

**Locations:**

Aarhus University Hospital, Aarhus Denmark

Rigshospitalet, Copenhagen, Denmark

Odense University Hospital, Odense, Denmark

|  |
| --- |

**Projekt group/investigators:**

Aarhus University Hospital, Aarhus Denmark:

Thomas Decker Christensen

Hans K. Pilegaard

Henrik Vad

Søren Pedersen

Anne-Mette Hvas

Vibeke Lauersen

Rigshospitalet, Copenhagen, Denmark:

Kåre Hornbech

Nora E. Zois

Odense University Hospital, Odense, Denmark:

Peter B. Licht

Mads Nybo

**Funding:**

Private and public foundations will be applied for economic support (app. 100,000 USD)

**Budget**

Has been thoroughly described in an appendix (app. 100,000 USD)

**References:**

1. Jakobsen, E., et al., *Data from a national lung cancer registry contributes to improve outcome and quality of surgery: Danish results.* Eur.J.Cardiothorac.Surg., 2009. 35(2): p. 348-352.

2. Rueth, N.M. and R.S. Andrade, *Is VATS lobectomy better: perioperatively, biologically and oncologically?* Ann Thorac Surg, 2010. 89(6): p. S2107-11.

3. Papa, M.L., et al., *Thromboelastographic profiles as a tool for thrombotic risk in digestive tract cancer.* Exp.Oncol., 2007. 29(2): p. 111-115.

4. Attaran, S., P. Somov, and W.I. Awad, *Randomised high- and low-dose heparin prophylaxis in patients undergoing thoracotomy for benign and malignant disease: effect on thrombo-elastography.* Eur.J.Cardiothorac.Surg., 2010. 37(6): p. 1384-1390.

5. Sorensen, B., et al., *Whole blood coagulation thrombelastographic profiles employing minimal tissue factor activation.* J.Thromb.Haemost., 2003. 1(3): p. 551-558.

6. Fenger-Eriksen, C., et al., *Fibrinogen substitution improves whole blood clot firmness after dilution with hydroxyethyl starch in bleeding patients undergoing radical cystectomy: a randomized, placebo-controlled clinical trial.* J.Thromb.Haemost., 2009. 7(5): p. 795-802.

7. Collins P.W., et al., *Global tests of haemostasis in critically ill patients with severe sepsis syndrome compared to controls*. Br J Haematol. 2006 Oct;135(2):220-7.
